# Acetazolamide for the Management of Diuretic-Induced Chloride Depletion Alkalosis: A Systematic Review

**DOI:** 10.3390/jcm14041041

**Published:** 2025-02-07

**Authors:** Fahad S. Alkhuzaee, Namareq F. Aldardeer, Omar A. Althobaiti, Abdulrahman S. Aljuaid, Abdulmajeed M. Alshehri

**Affiliations:** 1 King Abdullah Medical City, Makkah 24246, Saudi Arabia; alkhuzaee.f@kamc.med.sa; 2 King Faisal Specialist Hospital and Research Center, Jeddah 23433, Saudi Arabia; aldardeern@hotmail.com; 3 King Abdullah Medical Complex, Jeddah 23816, Saudi Arabia; dr.omaralthobaiti@gmail.com; 4 King Abdulaziz University Hospital, Jeddah 21589, Saudi Arabia; aljuaid77@gmail.com; 5 Department of Pharmacy Practice, College of Pharmacy, King Saud bin Abdulaziz University for Health Sciences, Riyadh 11481, Saudi Arabia; 6 King Abdulaziz Medical City, National Guard Health Affairs, Riyadh 11426, Saudi Arabia; 7 King Abdullah International Medical Research Center, Riyadh 11481, Saudi Arabia

**Keywords:** acetazolamide, chloride depletion alkalosis, metabolic alkalosis, systematic review

## Abstract

**Background**: Acetazolamide is a carbonic anhydrase inhibitor that inhibits proximal sodium bicarbonate reabsorption, thus increasing urinary bicarbonate excretion. Despite its widespread distribution in the body and beneficial effects on alkaline diuresis, its efficacy and the optimal dosage and duration of acetazolamide in treating metabolic acidosis remain areas of uncertainty. This review aims to assess the effectiveness of acetazolamide in treating chloride depletion alkalosis, mainly induced by diuretics, through a systematic evaluation of clinical research data. **Methods**: A comprehensive search was conducted on PubMed and Embase. This review included randomized controlled trials, observational studies, and case reports. Data extraction included dose, route, frequency, indication, duration of therapy, patient demographics, and outcomes. **Results**: A comprehensive search strategy identified 107 studies, of which 7 met the inclusion criteria after full-text review. The reviewed studies encompassed two randomized clinical trials, one case–control study, and three case reports, collectively involving 111 patients with metabolic alkalosis. The studies revealed varied outcomes regarding the efficacy of acetazolamide in treating metabolic alkalosis induced by diuretics. While some trials demonstrated significant improvements in serum bicarbonate levels and acid–base balance, others found no statistically significant reduction in the duration of mechanical ventilation. Case reports highlighted the successful use of acetazolamide in diverse patient populations, including pediatric patients with heart disease and individuals with chronic obstructive pulmonary disease. **Conclusions**: Acetazolamide holds promise in addressing chloride depletion alkalosis. However, a targeted clinical trial investigating its effectiveness in diuretic-induced metabolic alkalosis must strengthen the existing knowledge base.

## 1. Introduction

Acetazolamide is a carbonic anhydrase inhibitor that primarily inhibits proximal sodium bicarbonate reabsorption, thereby increasing urinary bicarbonate excretion. Its mechanism of action makes the medication the first line of solution for metabolic alkalosis [1]. Carbonic anhydrase is widely distributed in the body and catalyzes the kidney’s reaction, promoting hydrogen ion loss and the reabsorption of bicarbonate. Inhibition of carbonic anhydrase permits more of the filtered bicarbonate to be lost in the urine, resulting in alkaline diuresis. Carbonic anhydrase also optimizes the release of carbon dioxide (CO_2_) from muscle tissue, blood CO_2_ transport, and delivery of CO_2_ to alveolar gas.

High doses of diuretics can lead to metabolic alkalosis, resulting in diuretic resistance [2]. In 1965, Cannon and his colleagues established the concept of “contraction alkalosis” in the context of loop diuretics. They suggested that distal H + excretion with renal acid loss and rapid extracellular fluid (ECF) volume contraction at constant extracellular bicarbonate leads to metabolic alkalosis. It was observed that chloride loss influences bicarbonate reabsorption [3]. In patients with heart failure (HF), electrolytes are very important to monitor as they play a significant role in the management of fluid overload. In addition to sodium, growing evidence suggests that chloride may serve as a prognostic factor in patients with HF [4]. Moreover, the sodium-to-chloride (Na:Cl) ratio has emerged as a potential prognostic factor for mortality in HF patients, as an elevated Na:Cl ratio has been associated with an increased risk of mortality in this population [4].

Acetazolamide is limited by its transient effects and the development of metabolic acidosis with long-term use. However, acetazolamide can correct the significant metabolic alkalosis that occasionally occurs with loop diuretic therapy [5]. Even thiazide medications may cause profound metabolic alkalosis, which acetazolamide could be used to treat [6]. The mechanism that causes this type of alkalosis stems from chloride excretion when a considerable increase in bicarbonate excretion compensates for deficient chloride excretion. The pH will rise eventually [7].

Nevertheless, because of the lack of scientific agreement on the optimum dose and duration of acetazolamide for patients with metabolic alkalosis, the practices employed vary between healthcare providers, and, as a result, it is unlikely that beneficial outcomes are consistently achieved to avoid increased intensive care unit (ICU) mortality and morbidity [8] and the associated adverse effects on cardiovascular, pulmonary, and metabolic function [9]. Consensus on the above parameters must be established to achieve consistent results in resolving this disorder. However, this is currently impossible, as no systematic reviews are available. This review aims to evaluate the efficacy of acetazolamide in treating chloride depletion alkalosis with a focus on clinical research data.

## 2. Methods

### 2.1. Search Strategy, Study Selection, and Data Extraction

A comprehensive search was conducted across electronic databases such as PubMed, Embase, and Web of Science. The following keywords were used: acetazolamide, alkalosis, diuretics. All abstracts and titles were screened to identify studies examining the use of acetazolamide in diuretic-induced alkalosis. Exclusion criteria included non-human studies, articles not written in English, and articles with no full text available. Reference lists were reviewed to identify additional relevant publications. Non-human or non-English-language articles were excluded. The identified articles were reviewed and summarized by two authors. Data including dose, route, frequency, indication, duration of therapy, cause of disorder, and primary and/or secondary outcomes were extracted and collected from the included articles. Patient-related data included age, gender, and patient outcome. All data were analyzed using descriptive statistics. This systematic review was not registered in any systematic review registry; however, it was conducted according to the Preferred Reporting Items for Systematic Reviews and Meta-Analyses (PRISMA) reporting guidelines [10].

### 2.2. Quality Assessment and Risk of Bias

**Figure 1:**
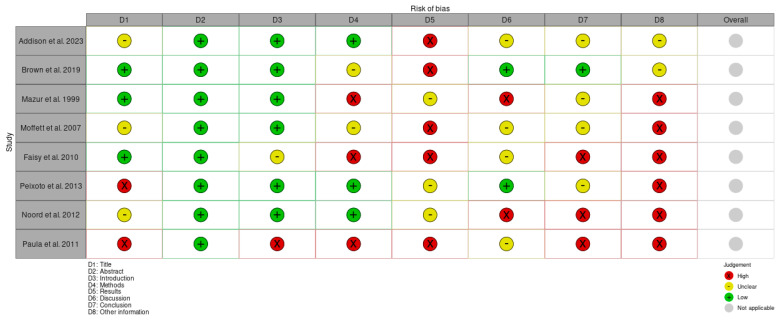
Risk of bias of the included studies [11,12,13,14,15,16,17,18].

**Figure 2:**
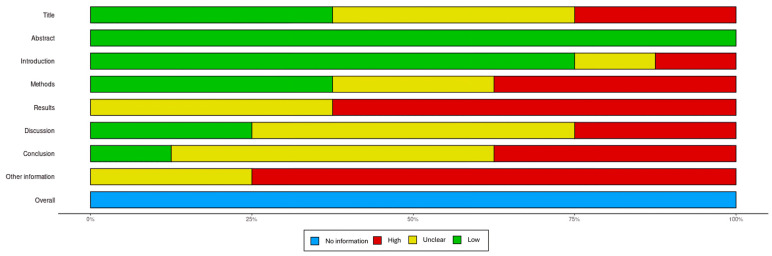
Visual representation of the risk of bias evaluation for different sections of studies.

To standardize the interpretation of the Consolidated Standards of Reporting Trials (CONSORT) guidelines for randomized trials, Strengthening the Reporting of Observational Studies in Epidemiology (STROBE) guidelines for observational studies, and Consensus-based Clinical Case Reporting Guideline Development (CARE) guidelines for case reports, explicit criteria for each item on the respective checklists were pre-established by three authors in accordance with the CONSORT, STROBE, and CARE explanation and elaboration documents. Based on these definitions, detailed data sheets were developed, containing questions for all items on the CONSORT, STROBE, and CARE checklists (Table 1). Depending on the study design, the appropriate checklist was utilized to evaluate the quality and adherence to reporting standards. The CONSORT checklist was applied to randomized controlled trials, the STROBE checklist was used for observational studies, such as cohort and case–control studies, and the CARE checklist was employed for case reports. Each included study was independently assessed. The assessors reviewed the studies and recorded their evaluations for each checklist item. Disagreements between assessors were resolved through discussion until consensus is reached. Adherence to the criteria was assessed with a single question for each item when no subitems were present. For items with multiple subitems, each subitem was assessed individually, resulting in a total of 34 questions across all three guidelines. Responses to these questions were scored as follows: “Yes” (all requirements fulfilled) received a score of 2, “Partial” (some but not all requirements fulfilled) received a score of 1, “No” (not all requirements fulfilled) received a score of 0, and “Not Applicable” (NA) was recorded where relevant. Depending on the specific item, one or more checkpoints were implemented to ensure that all requirements were thoroughly evaluated, based on the respective CONSORT, STROBE, and CARE explanation and elaboration documents. Final scores for each study were aggregated across all items and expressed as a percentage of the maximum achievable score (Figure 1 and Figure 2).

**Table 1:** Methodological quality assessment of included studies.

Studies	Study Design	Quality Score	Intervention
Addison et al., 2023 [14]	Multicenter, retrospective cohort study	58.3%	IV versus PO acetazolamide for a total dose of 500 mg in first 24 h
Brown et al., 2019 [13]	Single-center pilot randomized controlled trial	71.4%	Single bolus dose of 500 mg acetazolamide
Mazur et al., 1999 [11]	Randomized, double-blind, placebo-controlled trial	48.5%	One dose of 500 mg or 250 mg acetazolamide every 6 h for four doses
Moffett et al., 2007 [15]	Retrospective cohort study	37.5%	5 mg/kg of acetazolamide over a 3-day course (7 patients received it through the oral route, while 21 received it through the intravenous route)
Faisy et al., 2010 [12]	Case–control study	32.7%	Single intravenous dose of acetazolamide (500 mg daily)
Peixoto et al., 2013 [18]	Case report	72.4%	IV acetazolamide 250 mg every 6 h for 24 h or as a single dose of 500 mg
Noord et al., 2012 [16]	Case report	60.3%	Case used acetazolamide (dose was not mentioned)
Prieto de Paula et al., 2011 [17]	Case report	36.2%	Acetazolamide 500 mg/day for 3 days

### 2.3. Data Synthesis and Analysis

Due to the anticipated variability among the included studies, conducting a meta-analysis was not feasible. Instead, the results were synthesized narratively. The findings were structured and presented according to the study designs and the interventions evaluated, emphasizing the endpoints and conclusions reported in each study.

## 3. Results

**Figure 3:**
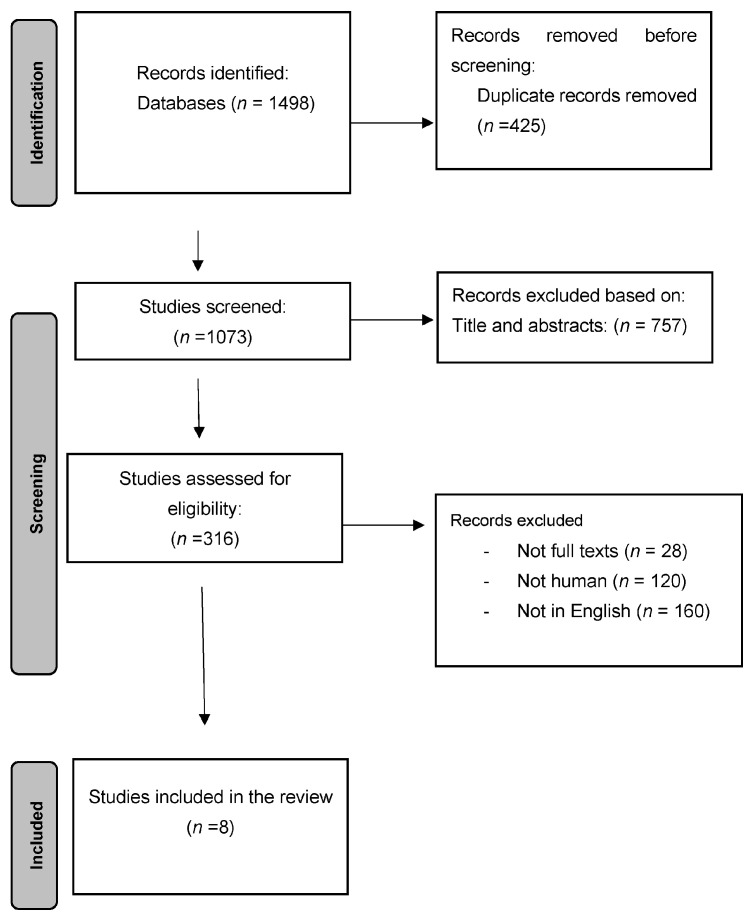
Flow diagram for selection of studies included in the systematic review.

A total of 108 articles were identified from the search strategy and reviewed by all authors. Twenty abstracts were identified by full-text review. After a full-text review, eight articles met the inclusion criteria (Figure 3). These included four clinical studies, one case–control study, and three case reports representing 80 critical patients who were mechanically ventilated, 28 pediatric patients, and 3 patients with metabolic alkalosis. The objectives of the included studies varied (Table 2).

The present systematic review summarizes the literature reporting diuretic-induced chloride depletion alkalosis. A total of 146 patients were identified. Only four studies were clinical trials (Table 3). The first clinical trial conducted was a randomized, double-blind, placebo-controlled trial that included 40 mechanically ventilated patients with metabolic alkalosis resistant to fluid or potassium therapy not receiving acetazolamide or sodium bicarbonate in the previous 72 h (arterial pH ≥ 7.48, HCO_3_^−^ ≥ 26 mEq/L). The study participants were stratified by last diuretic use and randomized to receive the following: acetazolamide IV, one dose of 500 mg, and acetazolamide IV, 250 mg, every 6 h for a total of four doses. The findings showed no difference between the two dosing regimens at any point over the study period for serum HCO_3_, serum K, or urine chloride endpoints. The results did not differ between diuretic- and nondiuretic-treated patients. Serum HCO_3_ remained significantly decreased in both treatment groups 72 h after the administration of the first acetazolamide dose (31.8 ± 4.9–25.3 ± 3.8 mEq/L, *p* < 0.0001 [250 mg × 4]; 31.9 ± 25.4–25.4 ± 3.6 mEq/L, *p* < 0.0001 [500 mg × 1]). The authors concluded that a single 500 mg dose of acetazolamide reverses chloride-resistant metabolic alkalosis in medical intensive care unit patients as effectively as multiple doses of 250 mg [11].

The second clinical trial was conducted to determine the effect of acetazolamide on the duration of invasive mechanical ventilation, where acetazolamide (500–1000 mg, twice daily) was administered intravenously in cases of pure and mixed metabolic alkalosis. The authors found that using acetazolamide, compared with placebo, did not result in a statistically significant reduction in the duration of invasive mechanical ventilation. Still, it showed daily changes in serum bicarbonate, and the number of days with metabolic alkalosis decreased significantly more in the acetazolamide group than in the placebo group. The authors of this study concluded that among patients with COPD receiving invasive mechanical ventilation, the use of acetazolamide, compared with placebo, did not result in a statistically significant reduction in the duration of invasive mechanical ventilation. However, the magnitude of the difference was clinically important, and it is possible that the study was underpowered to establish statistical significance [12].

The third clinical trial compared the metabolic effects of a single intravenous dose of acetazolamide in critically ill patients. It was a single-center pilot randomized controlled trial [conducted] at a tertiary adult intensive care unit (ICU). The participants were 26 adult ICU patients who were deemed to require diuretic therapy. A single dose of intravenous 500 mg acetazolamide was given, and the results showed that acetazolamide had decreased both plasma pH and HCO_3_ significantly (change in median serum pH, −0.01 [interquartile range, IQR, −0.04–0.01]; *p* = 0.01; change in median serum HCO_3,_ −2 mmol/L [IQR, −3 to 0]; *p* < 0.01) [13].

The most recent cohort study was a multicenter retrospective cohort study comparing IV versus PO acetazolamide use in patients with heart failure (HF) receiving at least 120 mg of furosemide with metabolic alkalosis (serum bicarbonate CO_2_ ≥ 32). The primary outcome was the change in CO_2_ on the first basic metabolic panel (BMP) within 24 h of the first dose of acetazolamide. IV acetazolamide was given to 35 patients, and PO acetazolamide was given to 35 patients. Patients in both groups were given a median of 500 mg of acetazolamide in the first 24 h. For the primary outcome, there was a significant decrease in CO_2_ on the first BMP within 24 h after patients received the IV acetazolamide (−2 [IQR: −2, 0] vs. 0 [IQR: −3, 1], *p* = 0.047). IV acetazolamide resulted in significantly decreased bicarbonate within 24 h of administration [14].

Several case reports were described regarding the subject matter, with a total number of 30 patients who were on diuretics before developing alkalosis. The first case report discussed using acetazolamide to treat hypochloremic metabolic alkalosis in pediatric patients with heart disease. The authors aimed to assess the safety and efficacy of a 3-day dosing regimen of acetazolamide in these patients. A total of 28 patients were included in the case report, with a median age of 2.5 months. The results showed that acetazolamide was safely used in pediatric patients with heart disease to lower serum bicarbonate and excess acid–base values and raise chloride values in hypochloremic metabolic alkalosis. There were no significant differences in electrolyte levels, blood urea nitrogen, or serum creatinine, except for a decrease in serum bicarbonate (36.2 ± 4.6 vs. 30.9 ± 4.5 mmol/L, *p* < 0.001) and an increase in serum chloride (91.1 ± 6.8 vs. 95.4 ± 6.2, *p* < 0.03). Excess acid–base values and pH also decreased during the therapy. No adverse effects were reported in any patients, and no changes in urine output were observed after the administration of acetazolamide. The study also found a positive correlation between the reduction in serum bicarbonate levels and the initial serum bicarbonate value [15].

The second case report discusses the case of a 62-year-old man who presented with severe alkalosis. The patient had a history of chronic obstructive pulmonary disease (COPD) and was taking medication for high blood pressure and inhalation drugs for COPD. The physical examination revealed hypertensive and pitting edema in the lower extremities. Lab results showed severe alkalosis (arterial blood gas during this previous visit showed pH 7.4, pCO_2_ 47 mmHg, and bicarbonate 28 mmol/L). The authors outlined some possible causes of the case’s alkalosis, including chloride and potassium depletion. It is revealed that the patient had been using a high dose of furosemide and consuming a large amount of licorice, which contributed to the metabolic alkalosis. Treatment for the patient included acetazolamide as part of the treatment regimen. This led to a normalization of lab values [16].

The third case report included two chronic respiratory acidosis cases coexisting with metabolic alkalosis. The first case presented with obesity–hypoventilation syndrome and the second with chronic obstructive pulmonary disease (COPD). In both instances, treatment with 500 mg per day of acetazolamide improved these cases, with one case able to stop home oxygen therapy [17].

Lastly, the fourth case report included a 68-year-old patient who was transferred to the intensive care unit due to atrial fibrillation and pulmonary edema. He developed progressive alkalemia induced by furosemide and received acetazolamide doses, which led to improvement in his metabolic derangements [18].

## 4. Discussion

Electrolyte and acid–base imbalances are commonly encountered in hospitalized patients, including those with heart failure (HF). HF patients are often on multiple medications that can impact electrolyte and acid–base balance due to various reasons, such as impaired renal function and activation of the renin–angiotensin–aldosterone system (RAAS), which can lead to sodium and water retention, potassium depletion, and metabolic alkalosis [5,19]. Furthermore, the use of diuretics can worsen electrolyte and acid–base disturbances, particularly chloride depletion and metabolic alkalosis [20]. However, acetazolamide can be beneficial in managing diuretic-induced chloride depletion alkalosis by promoting chloride reabsorption and bicarbonate excretion, leading to the correction of acid–base metabolic alkalosis [14]. Minimal alternatives to acetazolamide are available to treat metabolic alkalosis. Treating metabolic alkalosis is vital for the correction of hypovolemia and hypokalemia. However, there are some cautions that need to be considered before using acetazolamide, such as hyponatremia, advanced heart failure, and risk of acute kidney injury. Furthermore, acetazolamide is contraindicated in patients with severe chronic kidney injury due to risk of electrolytes imbalances.

The evidence presented in this review is encouraging for using acetazolamide, with three randomized clinical trials focused on metabolic alkalosis, two observation studies, and four case reports comprising most of the available data. However, it is important to note the heterogeneity in the included studies, which includes differences in study design, doses, route, duration of acetazolamide, and population. Still, there is insufficient evidence to demonstrate at what dose and route of acetazolamide is appropriate to treat such cases and what minimal duration is needed.

Our review is the first to review evidence for the efficacy of acetazolamide use in diuretic-induced metabolic alkalosis with a focus on a clinically relevant questions and specific intervention. Several limitations have been identified from the literature, including a small sample size, few clinical trials, heterogeneity in study population and outcomes that were not clinically directed. Moreover, diuretics were not specified in some studies. The absence of focused, high-quality studies investigating the outcomes of metabolic alkalosis is the central issue affecting this review. Although case reports were documented on this subject, cases may not be generalizable for implementation; causes or associations may have explanations other than what’s studied. Large randomized controlled trials are needed to assess the clinical benefit of acetazolamide use for diuretic-induced metabolic alkalosis. Moreover, the initiation of a clinical pathway to limit the use of acetazolamide to specific criteria with pre-specified doses is needed in clinical practice.

## 5. Conclusions

Given the evidence shown in this review, acetazolamide seems promising for correcting chloride depletion alkalosis. However, the existing evidence is limited by heterogeneity among studies and the lack of large clinical trials. Despite that, acetaldoxime is commonly used in clinical practice to manage metabolic alkalosis. A focused clinical trial on diuretic-induced metabolic alkalosis is needed to build a more robust evidence on optimal dosing strategies and patient outcomes.

## Figures and Tables

**Table 2:** Objectives of included studies/reports.

Studies	Objectives
Addison et al., 2023 [14]	Characterize dosing strategies and determine the effectiveness of intravenous and oral acetazolamide for patients with heart failure with diuretic-induced metabolic alkalosis.
Brown et al., 2019 [13]	Compare the physiological and biochemical effects of a single intravenous dose of furosemide or acetazolamide in critically ill patients.
Mazur et al., 1999 [11]	Compare two dosing regimens of acetazolamide for the reversal of metabolic alkalosis in mechanically ventilated patients with asthma or chronic obstructive pulmonary disease.
Moffett et al., 2007 [15]	Assess the safety and efficacy of a 3-day dosing regimen of acetazolamide in pediatric patients with heart disease and hypochloremia metabolic alkalosis.
Faisy et al., 2010 [12]	Evaluate the effects of a single daily dose of acetazolamide on metabolic alkalosis and respiratory parameters in weaning chronic obstructive pulmonary disease (COPD) patients from invasive mechanical ventilation.
Peixoto et al., 2013 [18]	NA ^1^
Noord et al., 2012 [16]	NA ^1^
Prieto de Paula et al., 2011 [17]	NA ^1^

^1^ NA; not applicable.

**Table 3:** Main characteristics of included studies.

Studies	Study Design	Patients (n)	Intervention	Endpoints	Diuretics Used
Addison et al., 2023 [14]	Multicenter retrospective cohort study	35 heat failure patients receiving diuretics	IV versus PO acetazolamide for a total dose of 500 mg in first 24 h	A significant decrease in CO_2_ on the first BMP within 24 h after patients received the IV acetazolamide (−2 [IQR: −2, 0] vs. 0 [IQR: −3, 1], *p* = 0.047)	Furosemide at least 120 mg
Brown et al., 2019 [13]	Single-center pilot randomized controlled trial	13 critically ill patients requiring diuretic therapy	Single bolus dose of 500 mg acetazolamide	Plasma pH, median (IQR) −0.01 (−0.04 to 0) Serum HCO_3_ (mmol/L), median (IQR) −2 (−3 to 0)	NA ^1^
Mazur et al., 1999 [11]	Randomized, double-blind, placebo-controlled trial	40 mechanically ventilated patients with metabolic alkalosis resistant to fluid or potassium therapy	One dose of 500 mg or 250 mg acetazolamide every 6 h for four doses	Serum HCO_3_ after 4 doses of acetazolamide 250 mg; 31.8 ± 4.9–25.3 ± 3.8 mEq/L, *p* < 0.0001 Serum HCO_3_ after 1 dose of acetazolamide 500 mg; 31.9 ± 25.4–25.4 ± 3.6 mEq/L, *p* < 0.0001	Loop diuretics ^2^
Moffett et al., 2007 [15]	Retrospective cohort study	28 pediatric patients	5 mg/kg of acetazolamide for the 3-day course (7 patients received it through the oral route, while 21 received it through the intravenous route)	Serum HCO_3_ (mmol/L) 30.9 ± 4.5	Furosemide dose of 3 mg/kg per day and ethacrynic acid 1.69 mg/kg daily
Faisy et al., 2010 [12]	Case–control study	26 intubated COPD patients with mixed metabolic alkalosis (serum bicarbonate 26 mmol/L and arterial pH 7.38)	A single intravenous dose of acetazolamide (500 mg daily)	Serum HCO_3_ baseline 34 mmol/L, serum HCO_3_ after acetazolamide 32 mmol/L (*p* < 0.01)	NA ^1^
Peixoto et al., 2013 [18]	Case report	A 68-year-old ICU patient	IV acetazolamide 250 mg every 6 h for 24 h or as a single dose of 500 mg	HCO_3_ (mEq/L) was 38.4 before starting acetazolamide and 35.8 when started on said medication, and after 3 days of use, the bicarbonate level (mEq/L) was 28.5	Loop diuretics ^2^
Noord et al., 2012 [16]	Case report	A 62-year-old patient	Case used acetazolamide (dose was not mentioned)	Bicarbonate was 54 mmol/L	Furosemide (dose was not mentioned)
Prieto de Paula et al., 2011 [17]	Case report	Two patients with metabolic alkalosis	Acetazolamide 500 mg/day for 3 days	Case 1: serum HCO_3_ −38.2 mmol/L to 25.1 mmol/L Case 2: serum HCO_3_ −35 mmol/L to 25.4 mmol/L	Case 1: Furosemide 120 mg IV daily Case 2: Furosemide 60 mg PO daily

^1^ NA; not available; ^2^ not specified. HCO_3_: bicarbonate; IQR: interquartile range; IV: intravenous; PO: oral.

## Data Availability

The datasets used and/or analyzed during the current study are available from the corresponding author upon reasonable request.

## Abbreviations

BMP	basic metabolic panel
CO_2_	carbon dioxide
COPD	chronic obstructive pulmonary disease
CDA	chloride depletion alkalosis
ECF	extracellular fluid
HCO_3_	bicarbonate
HF	heart failure
ICU	intensive care Unit
IV	intravenous
IQR	interquartile range
pCO_2_	partial pressure of carbon dioxide
PO	oral
*p*	*p*-value
